# Emerging Pharmacological Properties of Cholinergic Synaptic Transmission: Comparison between Mammalian and Insect Synaptic and Extrasynaptic Nicotinic Receptors

**DOI:** 10.2174/157015911798376343

**Published:** 2011-12

**Authors:** Steeve H Thany, Hélène Tricoire-Leignel

**Affiliations:** Laboratoire Récepteurs et Canaux Ioniques Membranaires (RCIM), UPRES EA 2647/USC INRA 2023, Université d’Angers, UFR Sciences. 2 Bd Lavoisier, 49045 Angers cedex, France

**Keywords:** Nicotinic acetylcholine receptors, pharmacology, insect, mammal, synapse.

## Abstract

Acetylcholine (ACh) is probably the oldest signalling neurotransmitter which appeared in evolution before the nervous system. It is present in bacteria, algae, protozoa and plants. In insects and mammals it is involved in cell-to-cell communications in various neuronal and non-neuronal tissues. The discovery of nicotinic acetylcholine receptors (nAChRs) as the main receptors involved in rapid cholinergic neurotransmission has helped to understand the role of ACh at synaptic level. Recently, several lines of evidence have indicated that extrasynaptically expressed nAChRs display distinct pharmacological properties from the ones expressed at synaptic level. The role of both nAChRs at insect extrasynaptic and/or synaptic levels has been underestimated due to the lack of pharmacological tools to identify different nicotinic receptor subtypes. In the present review, we summarize recent electrophysiological and pharmacological studies on the extrasynaptic and synaptic differences between insect and mammalian nAChR subtypes and we discuss on the pharmacological impact of several drugs such as neonicotinoid insecticides targeting these receptors. In fact, nAChRs are involved in a wide range of pathophysiological processes such as epilepsy, pain and a wide range of neurodegenerative and psychiatric disorders. In addition, they are the target sites of neonicotinoid insecticides which are known to act as nicotinic agonists causing severe poisoning in insects and mammals.

## 1 INTRODUCTION

**Fig. (1):**
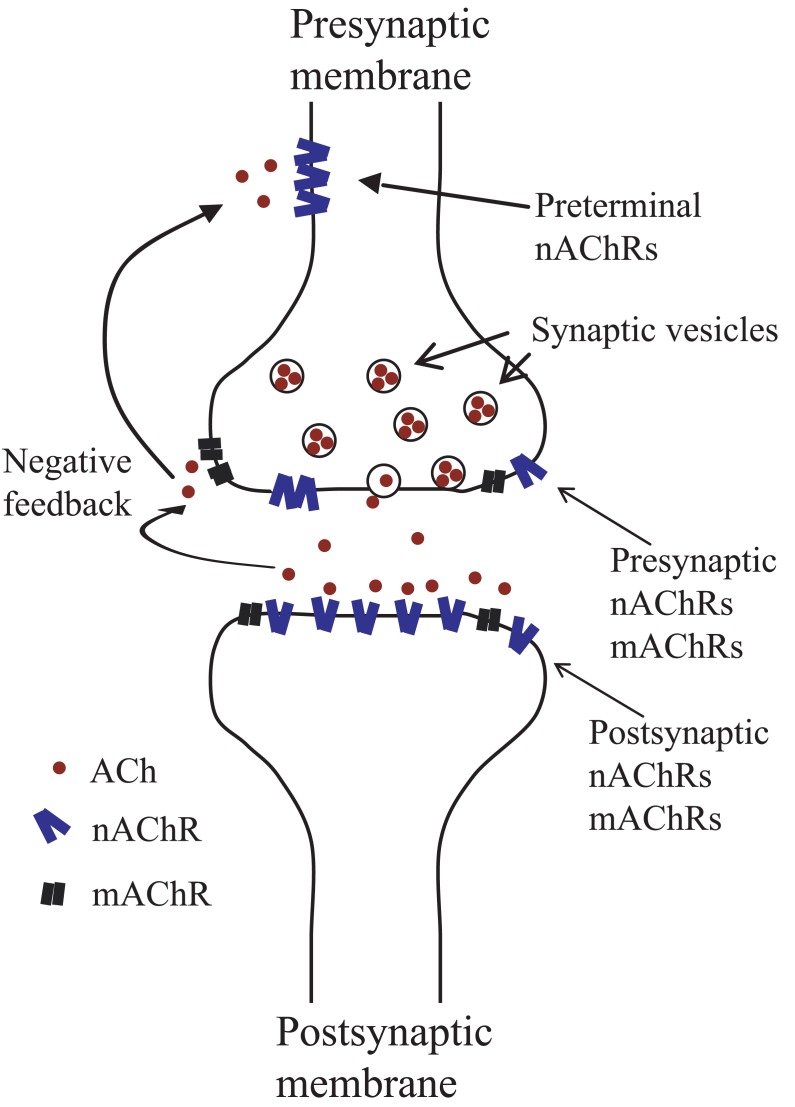
Putative locations of neuronal nicotinic acetylcholine receptors. ACh released from presynaptic vesicules may diffuse and activate presynaptic nAChRs and mAChRs resulting in a modulation of neurotransmitter release (negative feedback).

Cholinergic synaptic transmission plays a key role in the nervous system and biochemical changes at the synapse underlie some aspects of the higher brain function. It is known that acetylcholine (ACh) activates both nicotinic and muscarinic receptors in the insect and mammalian central nervous system (CNS). The nicotinic acetylcholine receptors (nAChRs) of the mammals fall into at least two subfamilies, occurring on muscle fibres at the neuromuscular synapses and on neurons in the peripheral ganglia and in the brain. In mammals, previous studies indicate that neuronal nicotinic receptors can be either presynaptic or postsynaptic (Fig. **1**) [1-4]. This assumption was confirmed by several studies showing that at the synaptic level, nicotinic receptors were associated with postsynaptic mediated excitation and membrane depolarization, while at presynaptic site, nAChRs activation could produce either excitation or inhibition indirectly through the release of endogenous transmitters or modulators. Consequently, they are likely to serve a number of physiological roles: they can act presynaptically to modulate neurotransmitter release and/or can function at extrasynaptic or synaptic sites to generate synaptic currents [5-10]. The availability of cDNA sequences coding for the neuronal nAChR subunits has made it possible to carry out studies on the distribution, [11-15] subunit composition and pharmacological profile of vertebrate nAChR subtypes [16-22]. Subsequently, several agonists and antagonists have been found that are selective, to varying degrees, for particular subtypes of these receptors.

In insects, it has also been demonstrated that nAChRs mediate rapid synaptic neurotransmission, and some properties of the involved channels have been described [23-25]. Building on this conjecture, it was suggested that nAChRs in insects might retain features of the ligand-binding primordial to ACh-activated channels and that native nAChRs may manifest a homo- and hetero-oligomeric subunits structure as few nAChR subunits have been isolated [26-29]. Although sharing many pharmacological and physiological properties with mammalian neuronal nAChRs, insect nAChRs have marked differences in their insecticide sensitivity, synaptic distribution and functional properties. Besides the classical postsynaptic receptors for ACh, nothing is known about the presynaptic nAChRs except that these receptors could mediate the release of ACh or modulate its release. This review will focus on the properties, mechanisms and pharmacological function of synaptic and extrasynaptic nicotinic receptors in insects. Our aim was to make a comparison of the cholinergic synaptic properties between insects and mammals. 

## 2 DIVERSITY OF INSECT AND MAMMALIAN NICOTINIC RECEPTOR SUBUNITS

Knowledge of the molecular organization of vertebrate nAChRs from neuromuscular junction and neurons has led to α subunits (α1 to α10) which have two vicinal cysteine residues equivalent to Torpedo Cys192 and Cys193 involved in ACh binding, and non-α subunits (β, γ, δ or ε) which are lacking this motif. The vertebrate muscle nAChR is a functional multisubunit receptor with a generic stoichiometry of 2α1, β, γ, δ or ε. The γ subunit is expressed embryonically and is replaced by ε subunit at synaptic junction in adult muscle [30]. Neuronal nAChRs constitute a more diverse family of receptors forming homo- and hetero-oligomeric channels [17, 19, 31-34]. The vertebrate nomenclature system is based on the subunits that compose those receptor types to the extent that information such as amino acid or nucleic acid sequences are available (e.g., the α2 subunit gene encodes the α2 subunit). Then, vertebrates possess at least 16 subunits while insects have more than 10 subunits [28,35,36] but their diversity is increased by RNA editing [27,37, 38]. 

Comparison between rat and human amino acid sequences typically reveals over 80% identity for a given nAChR subunit, allowing us to consider that *Rattus norvegicus* α2 subunit is similar to the *Homo sapiens* α2 one and in general to all vertebrate α2 subunits than *Rattus norvegicus* α7 subunit. The nomenclature currently used in insect is based on sequence apparition order which is reasonably insensible to subunit sequence homology between insect. For example the first peach potato aphid subunit discovered and named α1 is widely more homologous to the drosophila α2 subunit than the second identified peach potato aphid subunit named α2, which is homologous to the drosophila α1 one [28, 39]. Moreover in some cases, several nomenclatures exist for the same sequence subunit as in the drosophila: then, the drosophila second beta subunit [40] could be referred to as SBD, Dβ2 and nAcRβ-96A, this last nomenclature taking into account the gene’s chromosomal location as proposed by FlyBase (http://flybase.bio.indiana.edu). This does not provide a mechanism by which the nomenclature of genes and sequences from different insect species can be rationalised. Nevertheless, it was proposed that insect nAChRs, like mammalian neuronal nAChRs, are composed of five subunits, and can be pharmacologically subdivided into alpha-bungarotoxin (α-Bgt)-sensitive and – insensitive receptors [28] with the assumption that vertebrate α-Bgt-sensitive receptors form functional homo-oligomeric channels. 

## 3 INSECT POSTSYNAPTIC NICOTINIC RECEPTORS

Studies on the pharmacology of cholinergic synaptic transmission in insects have largely centred on the connections between afferents sensory neurons with interneurons or with motoneurons in several insects such as the cockroach *Periplaneta americana, *[41-45] and the tobacco hornworm, *Manduca sexta *[46]. It has been demonstrated that the postsynaptic neurons possess receptors which have an essentially nicotinic pharmacology: the most prominent effect of ACh is mediated by nicotinic receptors which generate fast excitatory postsynaptic potentials [47, 48] Several data have been obtained from cholinergic synaptic transmission at the cercal afferent-giant interneuron synapse in the cockroach sixth abdominal ganglion. Individual giant interneurons can be identified by their unique morphological characteristics and localization [42, 43, 49-51]. Iontophoretic injection of ACh onto the finer branches of giant interneurons resulted in a dose-dependent depolarization of a giant interneuron accompanied by a decrease in membrane resistance [43, 50, 51]. Following this experiment, profiles of ACh sensitivity were correlated with the distribution of the finer postsynaptic branches of a giant interneuron [52] as ACh-induced postsynaptic potentials were blocked by α-Bgt and also as the decline in amplitude of excitatory postsynaptic potentials (EPSPs) was associated with the response of giant interneuron to ACh [53, 54]. It was interesting to note that the minimal quantity of ACh required to generate a depolarizing response of giant interneuron (GI) 1 was estimated to 3.0 x 10^-17^ mol [51] which was comparable to values determined for vertebrates cholinergic synaptic transmission, close to 2.0 x 10^-18^ mol [55,56]. A similar action of ACh through activation of postsynaptic nAChRs could be also identified at the monosynaptic connections between the trochanteral hairplate afferents and motoneuron D_s_ of the metathoracic ganglion [57, 58], and in embryonic drosophila neurons* in vitro *[59]. 

## 4 PRESYNAPTIC ACETYLCHOLINE RECEPTORS: MUSCARINIC OR NICOTINIC RECEPTORS ?

In the conventional synapse, ACh released from the presynaptic vesicles, targets the postsynaptic nAChRs and opens ion gates. On the other hand, receptor sites were far from the release site. Terms of presynaptic receptors were defined as receptors at or near the nerve terminal (Fig. **1**) which can positively or negatively modulate transmitter release [8-10, 60]. In several studies, the sodium channel blocker, tetrodotoxin (TTX) has been used to define presynaptic nAChRs because in the presence of this toxin, action potentials arriving at the presynaptic terminals were eliminated [5, 61]. In the chick midbrain, accessory motoneurons extend their axons to the ciliary ganglion where they terminate in large calyces on ciliary cells [61-63]. Bath application of nicotine induced inward currents in the calyces capable of generating action potentials (APs). At presynaptic site, it was found that TTX blocked the APs but not the inward currents while α-Bgt blocked both. At postsynaptic level, α-Bgt was a partial antagonist and d-tubocurarine a full antagonist on nicotine-induced currents [61]. These results were consistent with the presynaptic action of nicotine through α-Bgt-sensitive α7 nAChR subtype. Similar activation of presynaptic α7 and non-α7 nAChRs has been identified in the rat sub-cortical regions, the frontal cortex and other vertebrate tissues [7,10,60]. 

The limited examples of excitatory or inhibitory ACh transmission through insect presynaptic nAChRs have prompted the hypothesis that the receptors serve other functions. Thus, intracellular microelectrode recording and ionophoretic application of carbamylcholine (CCh) were used to compare the cholinergic sensitivity of postsynaptic dendrites of giant interneuron 3 (GI 3) with that of presynaptic cholinergic axon of the lateral filiform hair sensory neuron (LFHSN) in the first-instar cockroach *Periplaneta americana *[64]. CCh responses of GI 3 and LFHSN were blocked by mecamylamine and d-tubocurarine but were not affected by muscarinic antagonists. From their studies, Blagburn and Sattelle [64] suggested that presynaptic nAChRs could be present in the axon membrane. They propose three possible locations of the ACh receptors on LFHSN: (1) postsynaptic to cholinergic input synapses onto the axon, (2) presynaptic to cholinergic output synapses made by the axon and (3) extrasynaptic [64]. Unfortunately, no clear evidence of insect presynaptic nAChRs has been demonstrated, and it was currently admitted that the postsynaptic neurons possess receptors that have an essentially nicotinic pharmacology mediating fast excitatory postsynaptic potentials (EPSPs) and that presynaptic receptors have only a muscarinic profile [46,65,66]. Evidence involving muscarinic receptors in synaptic function was obtained with the locust, *Locusta migratoria*, synaptosome preparation. It was found that these muscarinic receptors were similar to the mammalian M_2_ subtype receptor [67]. Electrophysiological studies performed on cockroach cercal nerve-giant fiber preparation have identified two distinct muscarinic receptor subtypes. The first subtype was present on the cercal afferent terminal with a pharmacological profile similar to the vertebrate M2 receptor, and the second muscarinic receptor was found on the membrane of the postsynaptic giant interneurons [65,68-70]. It was suggested that the presynaptic muscarinic receptors acted as autoreceptors regulating the release of ACh [65, 66, 68] while postsynaptic muscarinic receptors reduced the giant fiber spike threshold [71]. Comparable studies have been performed in other insects such as the locust *Schistocerca gregaria* [66] and the tobacco hornworm, *Manduca sexta* [46, 72]. The monosynaptic connection between sensory neurons and identified proleg motoneuron of the tobacco hornworm, *Manduca sexta* presents common characteristics with the cockroach cercal nerve-giant fiber. In fact, sensory neurons associated with a planta hair send an axon into the ganglion of the same segment where the afferent terminals make synaptic contact with interneurons and motoneurons such as proleg motoneuron (called PPR) [46, 72]. Trains of afferent activity cause a slow, long lasting depolarization that modulates PPR’s excitability. These EPSPs are mediated by mAChRs because they can be blocked by muscarinic antagonists and mimicked by agonists [46]. Thus, the responsiveness of motoneurons can be controlled by ACh through mAChRs [72]. 

## 5 NICOTINIC RECEPTORS EXPRESSED ON ISOLATED CELL BODIES

In the vertebrates, the availability of stable host cells expressing nAChR subtypes from humans or rats allowed further examination of nAChR pharmacology [73]. In fact, responses from native α7 nAChR demonstrate that the α7 subunit, when heterologously expressed in *Xenopus* oocytes, assembles into homopentameric ligand-gated ion channels that are cation-selective, rapidly desensitize and bind α-Bgt with high affinity [74, 75]. Thus, there was a strong correlation between native and expressed nAChR subtypes. Consequently, the minimum subunit combinations capable of forming functional receptors on expression systems have constrained views of the subunit composition of native neuronal nAChRs. Evidence obtained in host cells confirmed that pairwise combination of α2, α3 or α4 with β2 or β4 subunit generates heteromeric functional receptors [18, 76, 77] while α7 and α9 make an homomeric receptor [17,74,75]. One exception was that α10 subunit alone yielded no detectable functional receptors. However, co-injection with α9 subunit results in a functional nAChR subtype [17,19,78]. Consequently, two pharmacologically distinct nAChR subfamilies could be classified in the vertebrate: the α-Bgt-sensitive receptors which include the low-affinity homo-oligomer α7 receptor and the α-Bgt-insensitive receptors, which include the high-affinity hetero-oligomeric α_4_β_2_ receptor, widely distributed in the brain. 

The only expression of insect nAChR subunit in *Xenopus *oocytes was achieved with the locust,* Schistocerca gregaria*, α-like subunit αL1 [79, 80]. Compared to chick α7 responses, aL1 subunits were also able to form a homo-oligomeric channel which was blocked by α-Bgt and methyllicaconitine (MLA) but differed from α7 receptors in nicotine sensitivity and timecourse of evoked currents [80]. αL1 receptor is markedly less sensitive to nicotine than α7 receptor and current-voltage relationship was shifted towards more positive potentials, compared with α7 currents [80]. These results demonstrated that when experimental verification was possible, insect homomeric nAChRs could be pharmacologically distinct from vertebrate homomeric receptors. The last expression of insect nAChR subunit in heterologous systems was recently achieved by the cloning, sequencing and functional expression of a novel locust (*Schistocerca gregaria*) β subunit [81]. When co-expressed with a aL1 subunit, the pharmacology of the heteromeric receptor was indistinguishable from the one of the receptor based on a αL1 subunit alone [81]. On the other hand, oocytes co-injected with αL1 and Sgβ1 responded to nicotine with membrane depolarizations, although the peak depolarization amplitude did not differ from those responses with αL1 homomeric receptors. Because the pharmacological profile of the αL1/Sgβ1 heteromeric receptor refers to the αL1 homomeric receptor, Jones *et al*. [81] suggested that the Sgβ1 subunit was not able to form functional nAChR when co-expressed with αL1. This point was restrained by the finding that the vertebrate and insect β subunits are apparently incapable of forming functional nAChR alone. The most plausible explanation was given from vertebrate nAChRs obtained in transfected cell lines expressing either the human or rat nAChRs. It was observed that upregulation mechanisms differ from one expression system to another [82-86]. These mechanisms could alter functional expression of insect nAChRs in host cells. A second explanation could be that more complex subunit combinations are necessary because evidence for insect neuronal nAChR comprised of three subunits is accruing [87, 88]. In all cases, contrary to vertebrate nAChRs, the pharmacological profile of insect nAChRs associated with subunit composition was difficult to perform through host cells.

The initial experiments on native insect neurons were performed on the somata of neurons isolated on dorsal unpaired median (DUM) neurons of the grasshopper, *Schistocerca nitens* [89,90]. It has been shown that ACh-induced depolarizations were blocked by d-tubocurarine while α-Bgt failed to block the currents [90], suggesting a lack of α-Bgt-sensitive nAChRs. In line with this study, Lane *et al*. [91] demonstrated that α-Bgt blocked the depolarizing response to ionophoretic application of ACh onto the cell body membrane of the cockroach fast coxal depressor (D_f_) motoneuron but was completely ineffective in blocking the depolarizing action on DUM neurons [91]. These first results suggested a difference between nAChRs expressed on both DUM neurons and motoneuron D_f_ and likely that more than one type of nicotinic acetylcholine receptors was expressed in the isolated cells. Substantial investigation performed on cockroach neurons revealed the existence of α-Bgt-sensitive and –insentive nAChRs expressed on DUM neurons [92-94] and on neurons isolated from thoracic ganglia, two distinct α-Bgt-sensitive nAChR subtypes with distinct kinetic and pharmacological properties: a desensitized and a non-desensitized receptor named nAChD nAChRN, respectively [95]. 

## 6 INSECT NICOTINIC ACETYLCHOLINE RECEPTORS AND NON-CHOLINERGIC NEURONS

In non-cholinergic neurons such as dopaminergic, GABAergic and glutamatergic neurons, several vertebrate pre-synaptic nAChRs have been identified with a modulatory role on neurotransmission [96-100]. For example α7 nAChR subtype was involved in nicotine-mediated glutamate release and non-α7 subtype was involved in nicotine-mediated GABA release [96]. In addition, nAChR located on GABAergic interneurons reduced the GABA-mediated inhibition of dopamine (DA) release, thereby indirectly eliciting DA release [97, 98]. Moreover, it has been proposed that striatal muscarinic receptors (mAChRs) such as M2 and M4 receptors, by inhibiting ACh release from cholinergic interneurons, modify nAChR activity controlling DA release from dopaminergic neurons, suggesting that in other regions such as striatum and nucleus accumbens, there is evidence for presynaptic muscarinic receptors [101]. In all cases, presysnaptic muscarinic and nicotinic receptors have been identified in the vertebrate non-cholinergic neurons and their function was to modulate and/or inhibit neurotransmitter release.

**Fig. (2):**
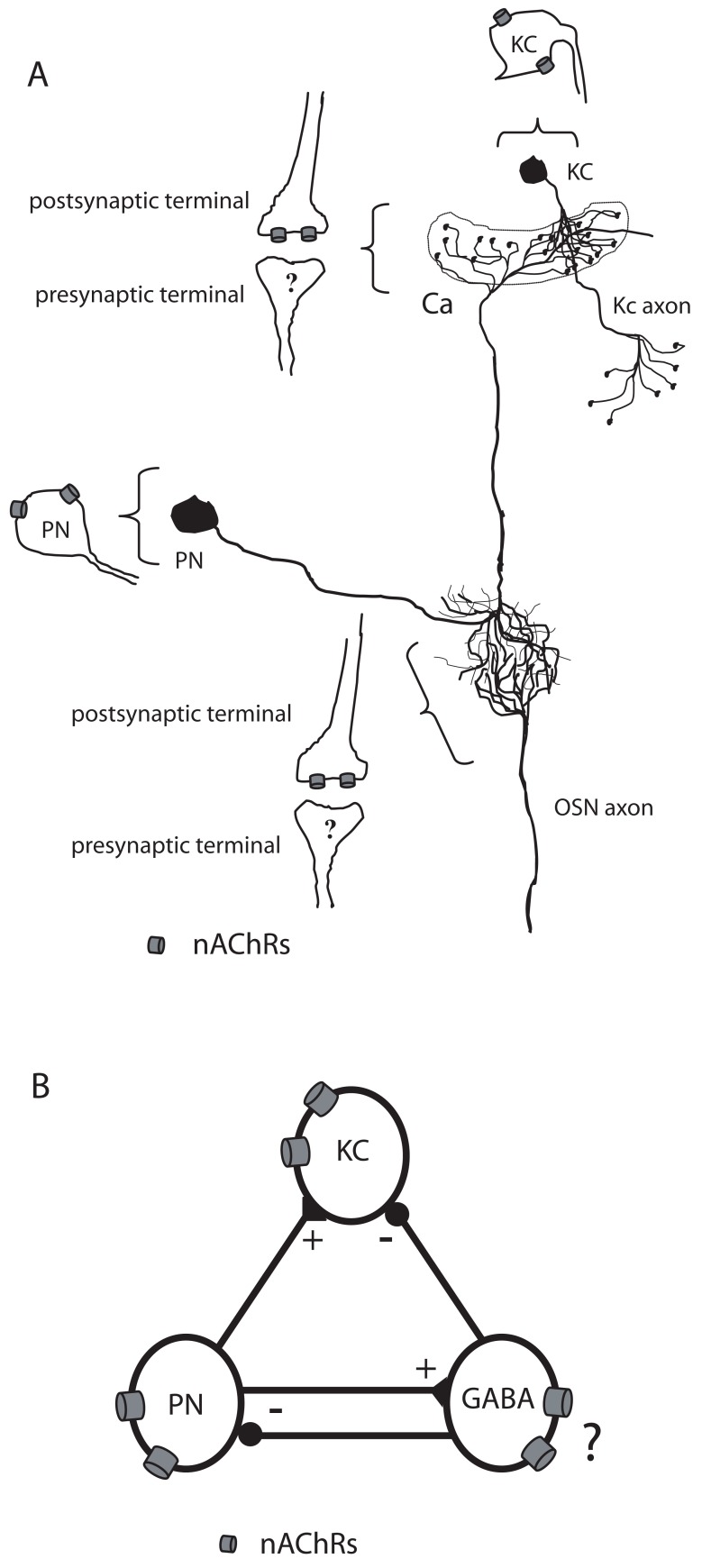
Putative locations of insect nicotinic receptors. (**A**) Olfactory sensory neurons (OSN) extend dorsally toward the brain and synapse in the glomerulus in the antennal lobe (AL). The cell body of projection neuron (PN) synapses with OSN in the antennal lobes and extend its axon dorsally to make synapses in the mushroom body calyx (Ca). Kenyon cells (KCs) axons target output neurons in the vertical and medial lobes of the mushroom bodies (also called α and β lobes). Following electrophysiological studies, nicotinic receptors could be localized on the cell bodies of KC, at postsynaptic sites between OSN and PN and also between PN and KC. (**B**) Synaptic connections between antennal lobe projection neurons (PN), Kenyon cells (KCs) and GABAergic inhibitory neurons within the mushroom body calyx neuropil. (?) indicates the putative locations of nicotinic receptors.

Despite that vertebrate nAChR subunits have been found on the soma of DA neurons [102] as well as on GABAergic terminals [103, 105], there was no clear evidence of specific nAChR subunit on insect non-cholinergic neurons. Physiological recordings in drosophila, locust and honeybee indicated that Kenyon cells (KCs) receive olfactory associative information directly from cholinergic projection neurons (PNs) located in the antennal lobes (ALs) and indirectly *via* GABAergic lateral horn neurons [106-109]. Excitatory information arrives from PNs located in the ALs while GABAergic lateral horn neurons, activated by odors, provided inhibitory inputs to the Kenyon cells (Fig. **2A**) [110,111]. Note that, within the lip region of the honeybee *Apis mellifera*, PNs make synapses onto GABAergic neurons which in turn send inhibitory synapses within PNs and KCs while GABAergic feedback neurons receive input in the mushroom bodies (MBs) and send their axons to the calyx lip region [110-112]. Further investigations in the drosophila demonstrated that KCs expressed α-Bgt-sensitive nAChRs which mediate fast excitatory synaptic transmission [113, 114]. Moreover, drosophila neuronal cultures in which cholinergic and GABAergic synapses are functionally formed revealed that DA suppressed cholinergic synaptic currents [115]. In fact, application of ACh and GABA on cultured KCs reveal that they express both nAChRs and GABA receptors [116-118]. These studies demonstrated that both nAChRs and GABA receptors were expressed in the same neurons. 

## 7 TOXICOLOGICAL MECHANISMS OF NEONICOTINOID ACTIONS ON INSECT AND MAMMALIAN nAChRs

Neonicotinoid insecticides represent a relatively new group of chemicals that includes imidacloprid, thiamethoxam, clothianidin and acetamiprid. They are highly efficient in suppressing the overwhelming majority of crop pests. It was known that the insecticidal activity of neonicotinoids is due to their agonist action on nicotinic receptors. At synaptic level, nenonicotinoid insecticides probably affect postsynaptic nAChRs [119, 120] less than presynaptic receptors. In fact, bath application of clothianidin on giant interneuron synapses induced a strong depolarization which was not blocked by muscarinic antagonists suggesting that its effect occurred on postsynaptic nicotinic receptors [120]. This synaptic effect could account for neonicotinoid symptoms described in the cockroach [121]. In fact, Tan *et al*. [121] have distinguished two neonicotinoid insecticide subgroups according to their effects. The first subgroup includes molecules resulting in strong excitation symptoms with uncoordinated quivering, hyper-excitability and rapid spontaneous movements of cockroaches, while there was no excitation symptoms in the second subgroup [121]. Although several electrophysiological studies have shown that neonicotinoid insecticides are likely to be low toxic in humans [122-124], several cases of acute poisoning have been associated with the development and the use of these insecticides [125, 126]. They share a similar mechanism of toxicity and therefore presenting patients have comparable symptoms such as respiratory, gastrointestinal, cardiovascular and central nervous system effects [126]. This apparent neonicotinoid toxicity, if associated with direct effect, suggests that the low affinity of these ligands to mammalians nAChRs must be clarified. In fact, we have recently found that thiamethoxam which was a poor agonist of insect nAChR on isolated cell bodies was able to generate a strong depolarization of the 6^th^ abdominal ganglion (personal observation). This effect suggested that there was a distinct effect of thiamethoxam on nAChRs expressed on isolated cell bodies compared to the one expressed at synaptic level.

## 8 CONCLUSIONS AND FUTURE PROSPECTS

The results of our initial efforts to compare insect and mammalian nAChR function suggested that according to pharmacological properties there is a strong correlation between insect and vertebrate nAChRs through their sensitivity to the weak agonist nicotine and the antagonist α-Bgt. In terms of antagonists, insect as well as vertebrate nAChRs were differentiated into two distinct nAChR subtypes: α-Bgt-sensitive and –insensitive receptors. However, there were two striking differences. The first was that expressed insect neuronal nAChRs of matching subunit composition can differ markedly from the vertebrate nAChRs in showing lower or higher sensitivity. The second was that presynaptic localization of insect nAChRs remains to be demonstrated (See Fig. **2B**), which is not the case for vertebrate nAChRs. 

The existence of multiple classes of insect nAChR found on isolated cell bodies does not preclude the existence of these receptors at synaptic level. As shown above, vertebrate nicotinic receptors sensitive to α-Bgt are present at presynaptic and postsynaptic levels. Although it was demonstrated that insect and vertebrate isolated cell bodies present α-Bgt-sensitive and –insensitive nAChRs, the former was not clear at insect presynaptic site. This lack of formal studies allows us to consider that only muscarinic receptors were expressed at insect presynaptic site and consequently account for the modulation of ACh release. In fact, works on cockroach and locust have provided evidence for postsynaptic function of nicotinic receptors and a presynaptic function of muscarinic receptors. It was noted that these postsynaptic nAChRs can be modulated by both muscarinic cholinergic and serotoninergic pathways [127, 128]. In this case, muscarinic pathway may act as a feedback mechanism to control ACh excitability to prevent excessive repeated depolarization but also regulate the actions of the inhibitory neurotransmitter GABA. Considering studies from vertebrate nAChRs showing the involvement of these receptors on presynaptic level and that in all the insects species investigated, a much higher density of CNS nicotinic receptors is detected compared to muscarinic receptors; therefore it would be conceivable to suggest that insect nAChRs could account for the same complex processes at presynaptic site. 

In conclusion, insects provide material suited to developmental and genetic approaches to the study of nicotinic receptors, their involvement in learning and memory processes and the toxic effect of active compounds such as neonicotinoid insecticides. This will help in providing new approaches to the chemical control of insect pests of agricultural, veterinary and medical importance. The development of novel nicotinic ligands for the treatment of cognitive deficits or as neonicotinoid insecticides depends on determining the differential roles of various nicotinic receptor subtypes. Key questions for future research: (1) Does insect presynaptic cholinergic neurons express nAChRs and if so, which subtypes? (2) What is the *in vivo* relevance of nAChRs expressed on isolated cell bodies while they did not receive efferent inputs? In fact, nAChRs have also been identified on the cell bodies of several insects [28] such as honeybee KCs and ALs [117, 118, 131, 132].
